# Smad4 in T cells plays a protective role in the development of autoimmune Sjögren's syndrome in the nonobese diabetic mouse

**DOI:** 10.18632/oncotarget.13437

**Published:** 2016-11-17

**Authors:** Donghee Kim, Jae Young Kim, Hee-Sook Jun

**Affiliations:** ^1^ Lee Gil Ya Cancer and Diabetes Institute, Gachon University, Incheon, Republic of Korea; ^2^ Department of Life Science, Gachon University, Seongnam, Gyeonggi-Do, Republic of Korea; ^3^ College of Pharmacy and Gachon Institute Pharmaceutical Science, Gachon University, Incheon, Republic of Korea; ^4^ Gil Medical Research Institute, Gil Hospital, Incheon, Republic of Korea

**Keywords:** Sjögren's syndrome, Smad4, TGF-beta, NOD mice, IL-17, Immunology and Microbiology Section, Immune response, Immunity

## Abstract

We investigated the role of Smad4, a signaling molecule of the TGF-beta pathway, in T cells on the pathology of Sjögren's syndrome (SS) in nonobese diabetic (NOD) mice, an animal model of SS. T cell-specific Smad4-deleted (Smad4^fl/fl,CD4-Cre^; Smad4 tKO) NOD mice had accelerated development of SS compared with wild-type (Smad4^+/+,CD4-Cre^; WT) NOD mice, including increased lymphocyte infiltration into exocrine glands, decreased tear and saliva production, and increased levels of autoantibodies at 12 weeks of age. Activated/memory T cells and cytokine (IFN-γ, IL-17)-producing T cells were increased in Smad4 tKO NOD mice, however the proportion and function of regulatory T (Treg) cells were not different between Smad4 tKO and WT NOD mice. Effector T (Teff) cells from Smad4 tKO NOD mice were less sensitive than WT Teff cells to suppression by Treg cells. Th17 differentiation capability of Teff cells was similar between Smad4 tKO and WT NOD mice, but IL-17 expression was increased under inducible Treg skewing conditions in T cells from Smad4 tKO NOD mice. Our results demonstrate that disruption of the Smad4 pathway in T cells of NOD mice increases Teff cell activation resulting in upregulation of Th17 cells, indicating that Smad4 in T cells has a protective role in the development of SS in NOD mice.

## INTRODUCTION

Sjögren's syndrome (SS) is a systemic chronic autoimmune disease that targets the exocrine glands, predominantly the salivary glands and lacrimal glands, resulting in xerostomia (dry mouth) and keratoconjunctivitis sicca (dry eyes) [1]. A focal lymphocytic infiltration of mainly CD4^+^ T cells is found in these glands in affected individuals [2, 3]. The nonobese diabetic (NOD) mouse is commonly used as a model of SS and spontaneously develops lymphocytic infiltration in exocrine glands with corresponding loss of secretory function [1].

Transforming growth factor-β (TGF-β) is a pleiotropic cytokine which regulates many biological processes including cell proliferation, differentiation, immune responses, and inflammation [4, 5]. TGF-β signaling is mediated through type I (TGF-βRI) and II (TGF-βRII) TGF-β receptors. Binding of TGF-β to TGF-βRI leads to phosphorylation of the signal transducers Smad2 and Smad3, which form a complex with Smad4 and translocate into the nucleus [6]. By interacting with other DNA-binding proteins, the Smad complex activates the transcription of TGF-β target genes [6]. TGF-β can affect all populations of leukocytes in a stimulatory or inhibitory manner depending on the context [7]. TGF-β has inhibitory effects on T cell and B cell proliferation, induces regulatory T cell differentiation and function [7, 8] and is important for the induction of Th17 cells [9, 10].

TGF-β1 mRNA and protein levels are elevated in the conjunctiva and salivary glands of SS patients [11, 12], suggesting that increased ductal expression of TGF-β may be important in the regulation of lymphoid infiltration and the proliferation of lymphocytes and ductal epithelium [13]. TGF-β1 null mice [14] or mice deficient for thrombospondin-1, a major physiological activator of latent TGF-β1 [15], develop ocular pathology and lacrimal gland inflammation. As well, abrogation of TGF-β signaling in T cells of normal mice leads to spontaneous T cell differentiation and autoimmune disease [16, 17].

Since TGF-β signaling is predominantly mediated by a Smad-dependent pathway [18], T-cell-specific deletion of Smad4 would be expected to block TGF-β signaling in T cells and to be phenotypically similar to T cell-specific TGF-βR-deficient mice [17, 19]. However, mice with T-cell-specific deletion of Smad4 are grossly normal without apparent T cell activation [20], although they develop gastrointestinal cancer [21]. In this study, we investigated the effects of Smad4 deletion in T cells on the development of SS in NOD mice and the mechanisms involved.

## RESULTS

### Development of SS-like ocular disease in Smad4 tKO NOD mice

**Figure 1:**
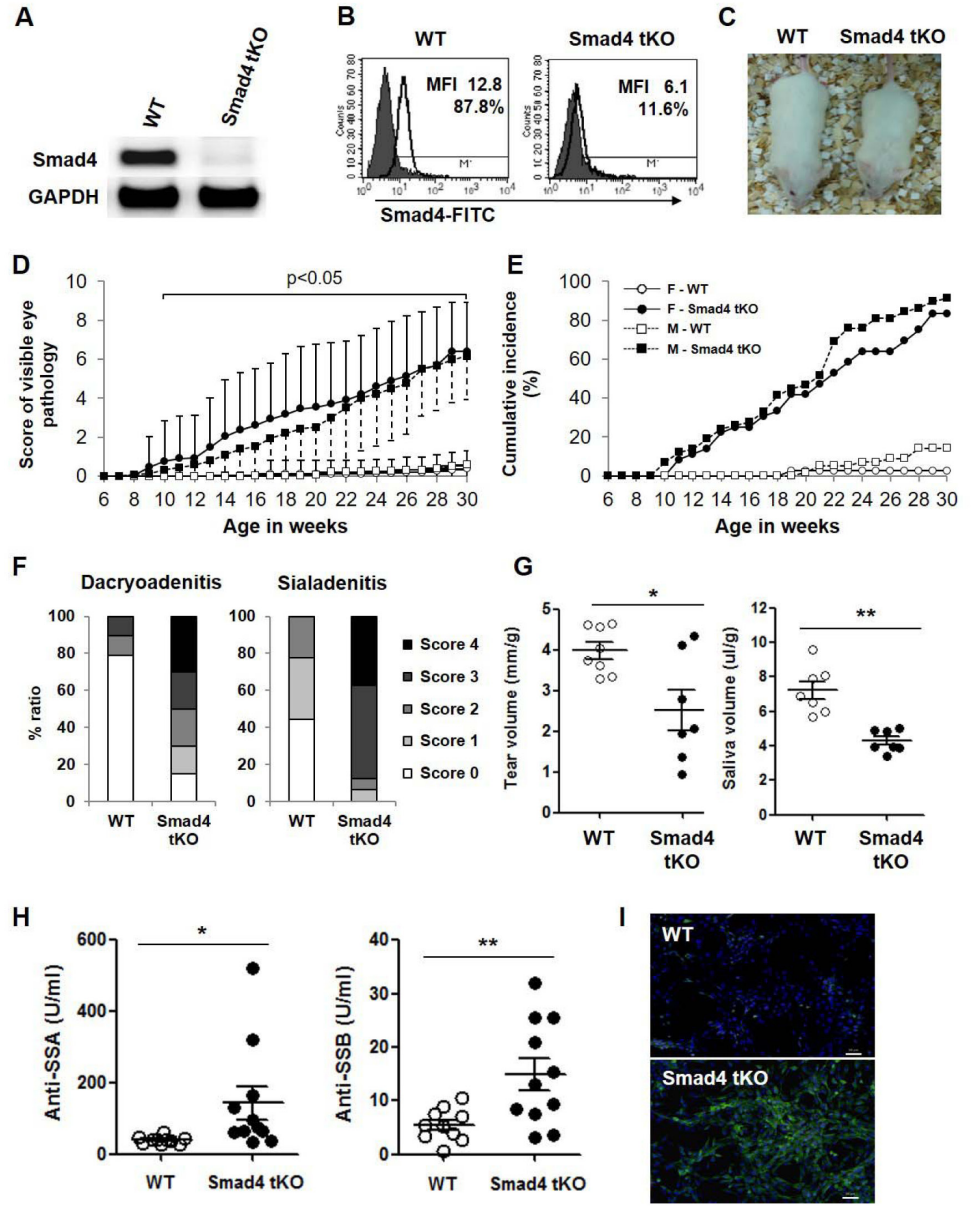
T cell-specific Smad4 deficiency increases the incidence of SS and accelerates disease onset in NOD mice **A.** Splenic T cells were isolated from 12-week old Smad4 tKO and WT NOD mice, and the expression of Smad4 mRNA was analyzed by RT-PCR. **B.** Splenocytes were stained with PE labeled anti-CD3 antibody, and then anti-Smad4 and FITC-anti-mouse antibodies. Smad4 expression was measured in CD3^+^ T cells by mean fluorescence intensity (MFI) using flow cytometry analysis. The solid line and gray field indicate the expression of Smad4 protein and isotype control, respectively. **C.** Gross appearance of WT and Smad4 tKO female NOD mice at 20 weeks of age. **D.** WT and Smad4 tKO NOD mice were scored for eye pathology (female Smad4 tKO, *n* = 36; WT, *n* = 56; male Smad4 tKO, *n* = 71; WT, *n* = 79). Values are means ± SD, *P* < 0.05, compared with the WT group; symbol legend as for E. **E.** Cumulative incidence of SS onset (combined score for both eyes over 4.0). **F.** Sections of lacrimal and salivary glands from 12-week old mice were stained with hematoxylin and eosin. The dacryoadenitis and sialadenitis was scored for focal inflammation as described in “Materials and Methods”. (**G**) Tear and saliva volumes and (**H**) auto-antibodies against SSA/Ro and SSB/La in sera from 12-week-old mice. (G and H) Each circle represents an individual mouse (*n* = 7-11/group). Values are means ± SD, *P < 0.05, **P < 0.01. **I.** NIH 3T3 cells were incubated with sera from 12-week-old mice and stained with anti-mouse IgG-FITC antibody and DAPI. Scale bar = 50 μm.

We generated NOD mice with a homozygous deletion of Smad4 in T cells (Smad4^fl/fl;CD4-Cre^, hereafter called Smad4 tKO) and wild type (Smad4^+/+;CD4-Cre^, hereafter called WT) NOD mice. We confirmed the absence of Smad4 mRNA (Figure 1A) and protein (Figure 1B) expression in T cells from Smad4 tKO NOD compared with WT NOD mice. Additionally, we checked the expression of Smad4 mRNA by qRT-PCR in various tissues, such as CD4^+^ T cells, B cells, eye and exocrine glands from WT NOD and Smad4 tKO NOD mice. Smad4 mRNA expression was almost not detected in CD4^+^ T cells of Smad4 tKO NOD mice compared with WT NOD mice, but was not different in non-T cell tissues including B cells, eye and exocrine glands (Supplementary Figure 1). The Smad4 tKO NOD mice showed symptoms of a wasting syndrome (Figure 1C); they were disheveled with hunched backs, swollen feet, dry tail skin, scruffy hair and died between 8 and 9 months of age. Interestingly, we visually observed eye disease in Smad4 tKO NOD mice. As ocular lesions progressed, the eyes of Smad4 tKO NOD mice blinked more frequently, the eyelids swelled, and eventually one or both eyes closed completely. According to disease scoring (Table 1), the eye pathology score was significantly higher in Smad4 tKO NOD compared with WT NOD mice (Figure 1D). Smad4 tKO NOD mice showed ocular disease from 10 weeks of age in both males and females, whereas the few WT NOD mice that did develop ocular disease showed the disease from 12 weeks in males and 19 weeks in females, suggesting very early onset of the ocular disease in Smad4 tKO NOD mice (Figure 1D). The cumulative incidence of ocular disease was 89% in female and 86% male Smad4 tKO NOD mice, whereas only 9% female and 11% male WT NOD mice showed ocular disease by 30 weeks of age (Figure 1E). We considered that this ocular disease is one specific symptom of SS. The pancreatic islet and submandibular glands of female NOD mice are more rapidly infiltrated with immune cells than male NOD mice [22]. However, cellular infiltration of the lacrimal glands is delayed in female mice; a study showed that ~25% of male lacrimal glands are infiltrated at 12 weeks of age, whereas age-matched female NOD mice still lack major signs of inflammation [22]. To clearly see differences between Smad4 tKO NOD and WT NOD mice, female mice, rather than male mice, were used to investigate the mechanisms of the SS acceleration by T cell-specific Smad4 deficiency.

**Table 1:** Definition of scoring by visible eye pathology

Score	Definition for each eye side	Examples
0	Normal eyelid, round eye shape	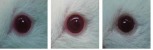
1	Slightly swollen eyelid, slightly distorted eye shape	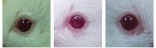
2	Swollen eyelid, reduced eye size (~ a half)	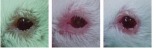
3	Swollen and injured eyelid, discharge in eye, reduced eye size (a half ~ a quarter)	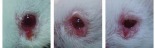
4	Below a quarter of eye size or closed eye	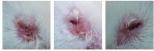

### Pathogenic markers of SS are increased in Smad4 tKO NOD mice

One of the key features of SS is lymphocytic infiltration of exocrine tissues, such as the lacrimal glands (dacryoadenitis) and salivary glands (sialadenitis). At 12 weeks of age, severe lymphocytic infiltration was observed in the lacrimal and salivary glands of Smad4 tKO NOD mice and this became more severe at 20 weeks of age, whereas relatively less infiltration was observed in these glands of WT NOD mice (Figure 1F and Supplementary Figure 2A and 2B). We measured tear and saliva production by pilocarpine stimulation at 12 weeks and 20 weeks of age. Tear and saliva volumes were significantly decreased in Smad4 tKO compared to WT NOD mice in 12 week-old mice (Figure 1G). At 20 weeks of age, saliva volume from Smad4 tKO NOD mice was further decreased and significantly lower than that of WT NOD mice, similar to the results of 12-week-old mice (Supplementary Figure 2C). However, tear volume was not different between Smad4 tKO and WT NOD mice at 20 weeks of age (Supplementary Figure 2C). These findings indicate that T cell-specific Smad4 deficiency resulted in an earlier functional impairment of the lacrimal and salivary glands as compared with WT NOD mice.

Another key feature of SS is the presence of circulating autoantibodies, specifically anti-SSA/Ro and anti-SSB/La. Smad4 tKO NOD mice produced significantly higher levels of anti-SSA/Ro and anti-SSB/La antibodies compared with WT NOD mice (Figure 1H). Consistent with this, IgG anti-nuclear antibodies were also increased in sera from Smad4 tKO NOD mice compared with WT NOD mice (Figure 1I).

**Figure 2:**
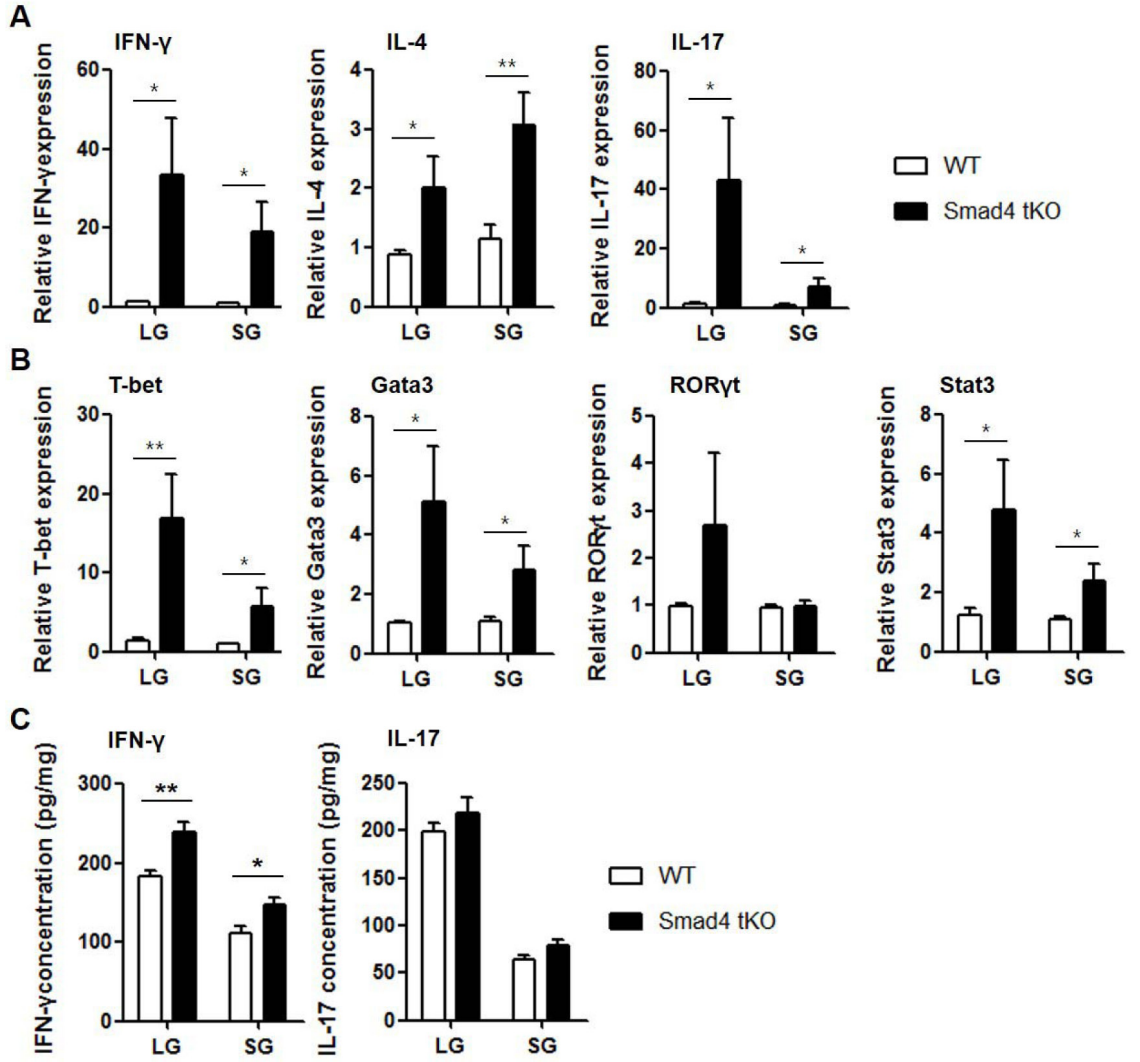
Expression of inflammatory cytokines is increased in exocrine glands from Smad4 tKO NOD mice (**A** and **B**) Total RNA and (**C**) tissue lysates were prepared from lacrimal and salivary glands of Smad4 tKO NOD and WT NOD mice at 12 weeks of age. The expression of mRNA for various cytokines (**A**) and transcription factors (**B**) was analyzed by qRT-PCR. Values are expressed as the relative fold-change as compared with WT. (**C**) IFN-γ and IL-17 production was measured by ELISA. Data are mean ± SD (*n* = 6~9/group). *P < 0.05, **P < 0.01.

We then examined the mRNA expression of cytokines and related transcription factors in the lacrimal and salivary glands by qRT-PCR. The expression of cytokines such as IFN-γ, IL-4, and IL-17 and these cytokine-specific transcription factors, such as T-bet for IFN-γ, Gata3 for IL-4 and signal transducer and activator of transcription (Stat)3 for IL-17, was significantly increased in both lacrimal and salivary glands from Smad4 tKO NOD mice compared with WT NOD mice (Figure 2A and 2B). When we examined the protein production of IFN-γ and IL-17 in the lysates of exocrine glands, we found that IFN-γ production was significantly higher in lacrimal and salivary glands from Smad4 tKO NOD mice compared with WT NOD mice (Figure 2C). All these results suggest that deletion of Smad4 in T cells leads to an acceleration of autoimmune SS.

### Cellular granularity and activated/memory T cells are increased in superficial cervical lymphocytes (SLCs) from Smad4 tKO NOD mice

**Figure 3:**
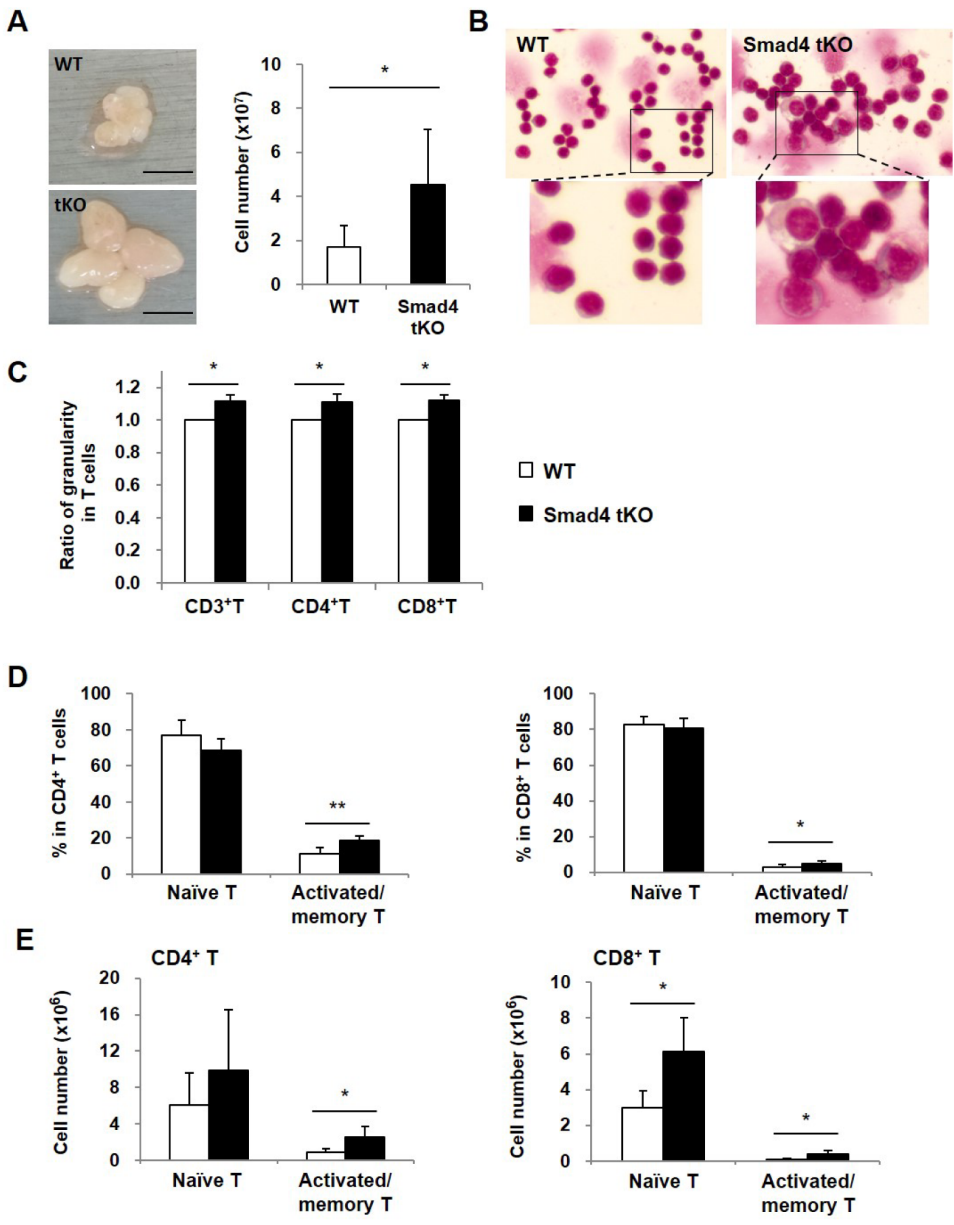
Cellular granularity and activated/memory T cells are increased in SLCs from Smad4 tKO NOD mice **A.** Superficial cervical lymph nodes were harvested and SLCs were isolated from 12-week old Smad4 tKO and WT NOD mice. And the cell numbers were measured. **B.** SLCs were stained with Wright-Giemsa and observed with a light microscope (×630). **C.** The cellular granularity of gated CD3^+^ T, CD4^+^ T and CD8^+^ T cells was measured by side scatter and normalized by mean fluorescent intensity in T cells from WT NOD mice. (D-E) SLCs were stained with anti-CD4 or anti-CD8, anti-CD44 and anti-CD62L antibodies and analyzed by FACS. Naïve cells were identified as having CD44^low^CD62L^high^ and activated/memory T cells as CD44^high^CD62L^low^ expression. The (**D**) proportion and (**E**) absolute cellular numbers of each cell population were determined as a percent of CD4^+^ and CD8^+^ gated T cells. Values are means ± SD (*n* = 6/group), *P < 0.05, **P < 0.01 compared with the WT group.

To investigate the mechanisms of early onset and higher incidence of SS, we first analyzed the superficial cervical lymph nodes as regional lymph nodes. In Smad4 tKO NOD mice, superficial cervical lymph nodes were larger than those from WT NOD mice (Figure 3A), and the total numbers of SLCs were increased by about 2.7 times in Smad4 tKO as compared with WT NOD mice (4.54 ± 1.58 × 10^7^ *vs* 1.69 ± 0.80 × 10^7^ cells) (Figure 3A).

T-cell large granular lymphocyte (LGL) leukemia is associated with multiple autoimmune conditions, such as rheumatoid arthritis [23]. To investigate whether SLCs of Smad4 tKO NOD mice have similar properties to LGLs, the size and granularity of SLCs were measured by light microscopy in Smad4 tKO and WT NOD mice at 12 weeks of age. We found that SLCs in Smad4 tKO NOD mice showed increased cell size and granularity compared with WT NOD mice (Figure 3B). Consistent with this, cellular granularity as measured by flow cytometry was also increased in the total T (CD3^+^ T), CD4^+^ T and CD8^+^ T cells from Smad4 tKO NOD compared with WT NOD mice (Figure 3C).

It was reported that naïve T cells are typically considered to be in a default state of quiescence, while memory T cells undergo basal proliferation and quickly exhibit effector responses when stimulated [24]. As the leukemic LGL cells are terminal effector memory T cells [25], we analyzed the proportion of naïve and effector memory T cells. In CD4^+^ T and CD8^+^ T cells, the proportion of naïve T cells (defined as CD44^low^CD62L^high^) was similar between Smad4 tKO NOD and WT NOD SLCs (Figure 3D); whereas the proportion of activated/memory T cells (defined as CD44^high^CD62L^low^) was significantly increased in Smad4 tKO NOD compared with WT NOD SLCs. Consistent with these results, the absolute numbers of CD4^+^ and CD8^+^ activated/memory T cells were significantly increased in SLCs from Smad4 tKO NOD compared to WT NOD mice (Figure 3E).

### IL-17 and IFN-γ production is increased in SLCs of Smad4 tKO NOD mice

**Figure 4:**
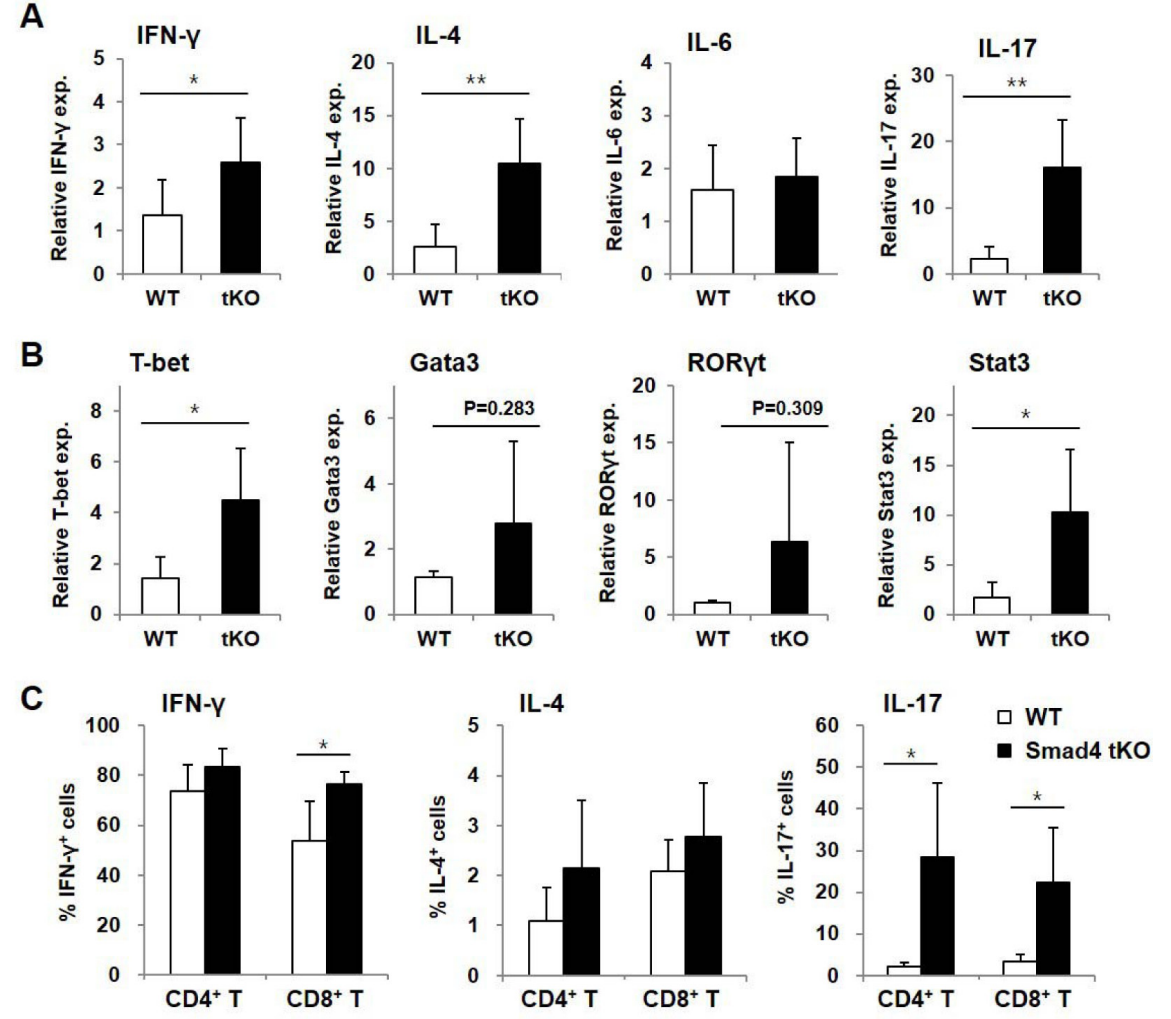
Inflammatory cytokine-expressing T cells are increased in SLCs from Smad4 tKO NOD mice **A.**-**B.** Total RNA was isolated from SLCs from Smad4 tKO and WT NOD mice at 12 weeks of age. The expression of mRNA for various (**A**) cytokines and (**B**) transcription factors was analyzed by quantitative real-time PCR. Values are expressed as the relative fold-change as compared with WT (*n* = 3-4/group). **C.** SLCs from Smad4 tKO and WT NOD mice at 12 weeks of age were stimulated with PMA (10 ng/ml) and ionomycin (500 ng/ml) for 12 h. The proportions of T cells producing IFN-γ, IL-4 and IL-17 were measured by FACS analysis (*n* = 5/group). Data are mean ± SD. *P < 0.05, **P < 0.01.

CD4^+^ effector T cells are known to migrate to target tissue sites of inflammation and rapidly produce both Th1 and Th2 cytokines after antigenic exposure [26]. To investigate the pathogenic roles of these cells, cytokines and transcription factors were measured in SLCs by qRT-PCR. mRNA transcripts of various inflammatory cytokines, such as IFN-γ, IL-4, and IL-17, were significantly increased in SLCs from Smad4 tKO NOD compared with WT NOD mice (Figure 4A). Expression of key transcription factors responsible for the expression of these cytokines, such as T-bet and Stat3, was also significantly increased in SLCs from Smad4 tKO NOD compared with WT NOD mice (Figure 4B). The expression of Gata3 and retinoic acid receptor-related orphan receptor (ROR)γt, however, did not differ between WT NOD and Smad4 tKO NOD mice.

To confirm the production of these cytokines, we analyzed cytokine-producing T cells by flow cytometry. The proportion of IFN-γ^+^CD8^+^ T cells and the proportion of both IL-17^+^CD4^+^ T and IL-17^+^CD8^+^ T cells were significantly increased in SLCs from Smad4 tKO compared with WT NOD mice (Figure 4C). The absolute numbers of these cytokine-expressing cells were also significantly increased in SLCs of Smad4 tKO NOD compared with WT NOD mice (data not shown). These results indicate that T cells from Smad4 tKO NOD mice are in a more active state compared with T cells from WT NOD mice.

### The proportion and function of Treg cells from Smad4 tKO NOD mice are not different from those of WT NOD mice

**Figure 5:**
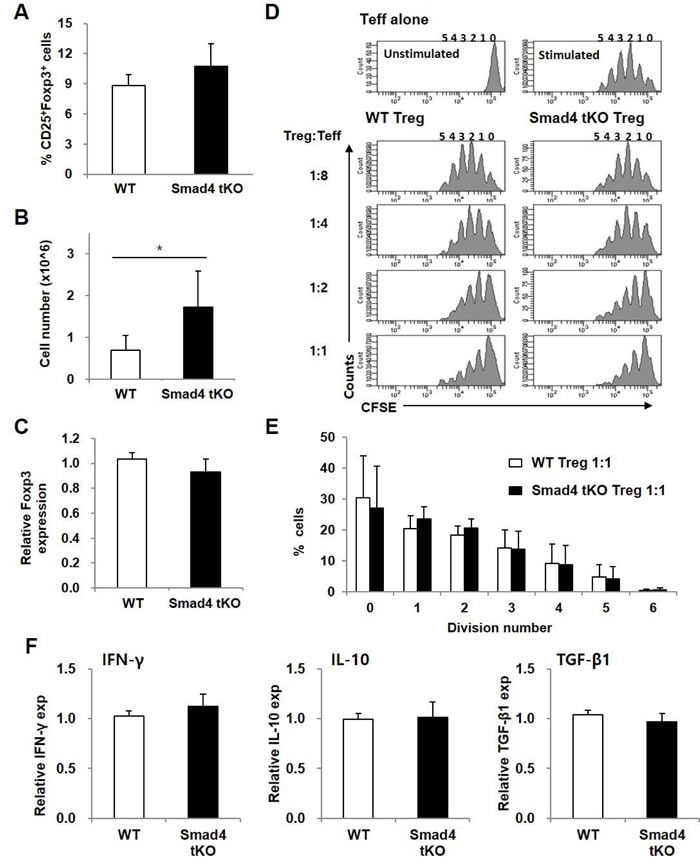
The proportion and suppressive function of Treg cells are maintained in Smad4 tKO NOD mice SLCs were isolated from Smad4 tKO and WT NOD mice at 12 weeks of age. (**A**) The proportion and (**B**) absolute number of Treg (CD4^+^CD25^+^Foxp3^+^ T) cells were determined by flow cytometry. **C.** The expression of Foxp3 mRNA was analyzed by qRT-PCR. **D.** Representative histogram plots of suppression assay of Treg cells. CFSE-labelled effector T cells (CD4^+^CD25^−^ T; Teff) from WT NOD mice were stimulated with anti-CD3/CD28-coated beads for 72 h in the presence of Treg (CD4^+^CD25^+^ T) cells from WT or Smad4 tKO NOD mice at various ratios (left margin). Proliferation of Teff cells was assessed by flow cytometry. **E.** The percentage of cells undergoing the indicated number of divisions when the Treg:Teff cell ratio was 1:1. Values are means ± SD (*n* = 3-4/group). (**F**) Total RNA was isolated from Treg cells and the expression of mRNA for IFN-γ, IL-10 and TGF-β was analyzed by qRT-PCR. Values are means ± SD (*n* = 8-9/group), *P < 0.05, **P < 0.01 compared with the WT group.

To investigate whether Treg cells in SLCs of Smad4 tKO NOD mice are defective in number or function, we analyzed the proportion, number and function of Treg cells. The proportion of Treg (CD4^+^CD25^+^Foxp3^+^ T) cells in SLCs was not different between Smad4 tKO NOD mice and WT NOD mice (Figure 5A), but the absolute number of Treg cells was significantly increased in Smad4 tKO NOD mice compared with WT NOD mice (Figure 5B). mRNA expression levels of Foxp3, a Treg-specific transcription factor, were similar between Smad4 tKO NOD and WT NOD mice (Figure 5C). Since functional defects in Treg cells can cause autoimmune disease [27], the immune-regulatory activity of Treg cells was evaluated by analyzing the proliferation of co-incubated Teff (CD4^+^CD25^−^ T) cells from WT NOD mice. The suppressive activity of Treg cells was similar between Smad4 tKO and WT NOD mice (Figure 5D, 5E). When we examined the expression of IFN-γ, IL-10 and TGF-β in Treg cells by qRT-PCR, the expression of these cytokines was similar between Smad4 tKO NOD and WT NOD mice (Figure 5F).

### Teff cells from Smad4 tKO NOD mice show reduced sensitivity to Treg cells and TGF-β

**Figure 6:**
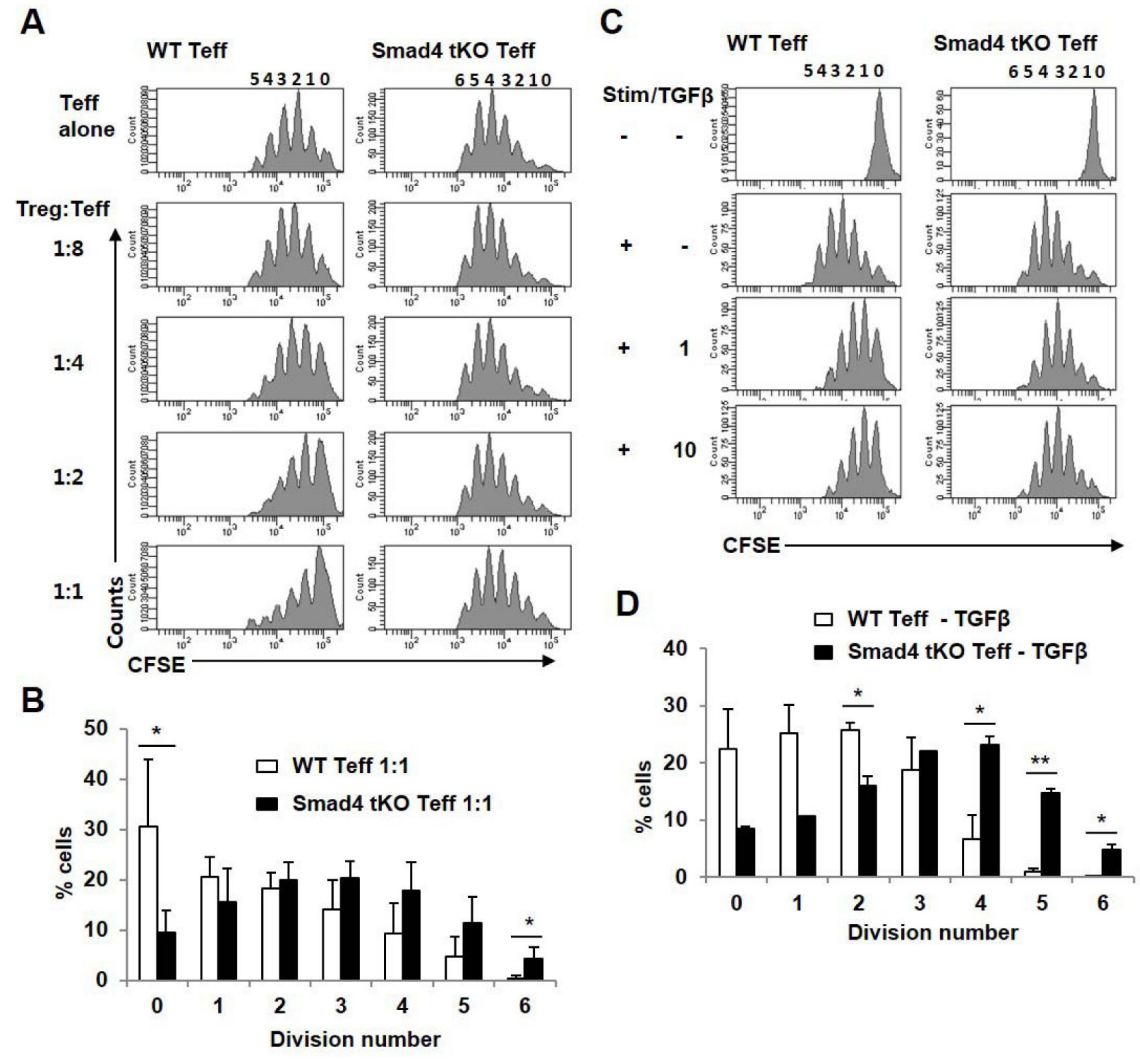
Teff cells from Smad4 tKO NOD mice have restricted sensitivity to Treg cells **A.** CFSE-labelled Teff cells from WT or Smad4 tKO NOD mice were stimulated (Stim) with anti-CD3/CD28-coated beads for 72 h in the presence of Treg cells from WT NOD mice at various ratios (left margin). Proliferation of Teff cells was assessed by flow cytometry. **B.** The percentage of cells undergoing the indicated number of divisions when the Treg:Teff cell ratio was 1:1. **C.** CFSE-labelled Teff cells were stimulated (Stim) with anti-CD3/CD28-coated beads for 72 h with or without TGF-β1 (1 or 10 ng/ml) and analyzed by flow cytometry. **D.** The percentage of cells undergoing the indicated number of divisions when exposed to 10 ng/ml of TGF-β1. Values are means ± SD (*n* = 3-4/group), *P < 0.05, **P < 0.01.

Since there was no difference in proportion or function of Treg cells between Smad4 tKO NOD and WT NOD mice, we investigated whether there is a difference in responsiveness of Teff cells to Treg cells. Teff cells from Smad4 tKO NOD and WT NOD mice were incubated with Treg cells from WT NOD mice, and the proliferative responses were examined. Treg cells suppressed WT Teff cell proliferation more than Smad4 KO Teff cell proliferation, suggesting that Teff cells of Smad4 tKO NOD mice were resistant to suppression by Treg cells compared with WT NOD mice (Figure 6A). Even when the ratio of Treg:Teff cells was 1:1, Teff cells from Smad4 tKO NOD mice actively divided (Figure 6B). Treg cells secrete inhibitory cytokines such as IL-10 and TGF-β, which directly inhibit Teff cell activation and effector function [28], therefore we analyzed the inhibitory effect of TGF-β on T cell proliferation. The proliferative response to anti-CD3/28 stimulation was significantly lower in Teff cells from WT NOD mice as compared with Teff cells from Smad4 KO NOD mice when incubated with TGF-β (Figure 6C, 6D).

### Teff cells from Smad4 tKO NOD mice show reduced Foxp3 expression and increased IL-17 expression under iTreg skewing conditions

**Figure 7:**
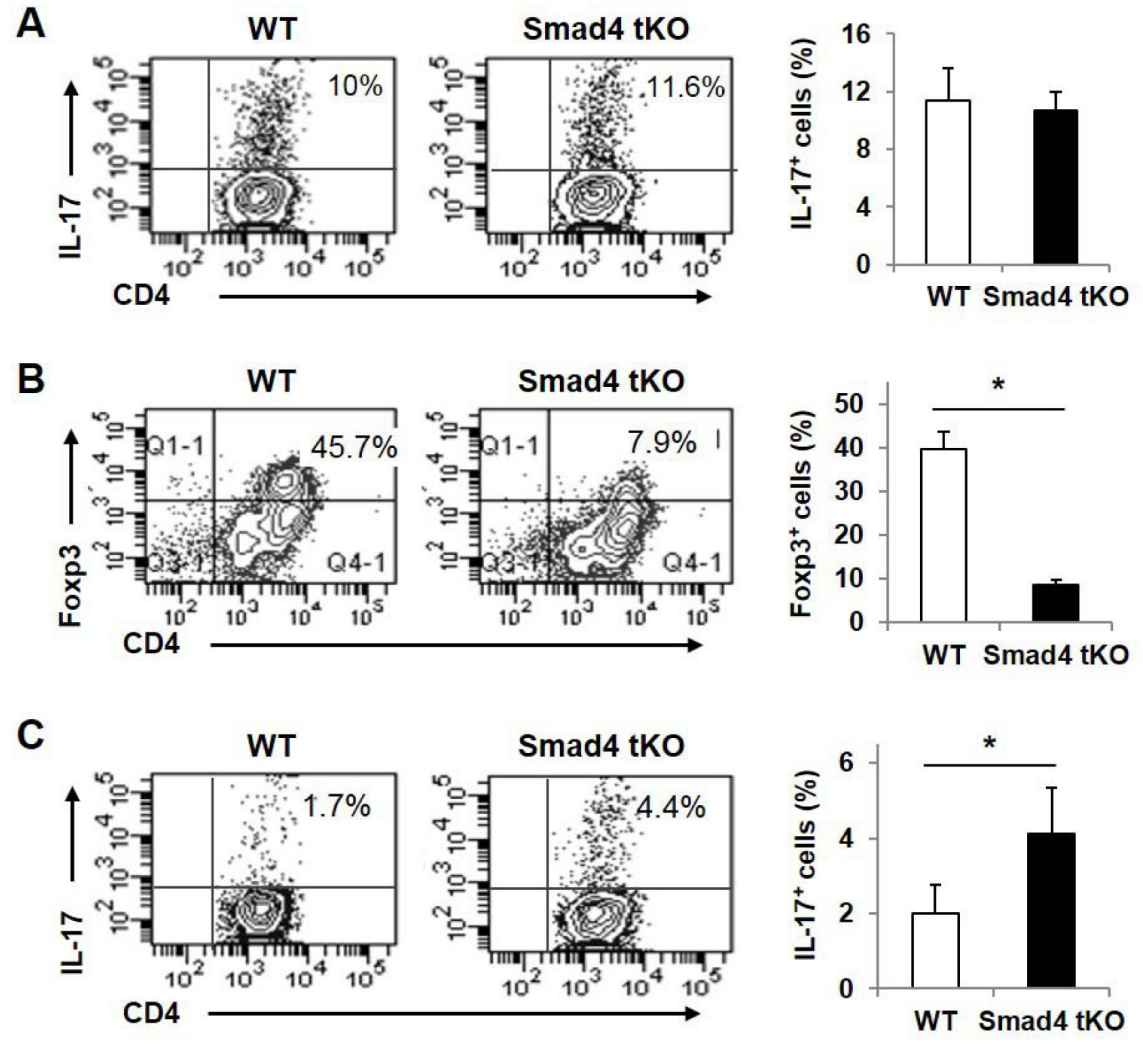
Teff cells from Smad4 tKO NOD mice show reduced Foxp3 expression and increased IL-17 expression under iTreg skewing conditions CD4^+^CD25^−^ Teff cells were obtained from SLCs of WT and Smad4 tKO NOD mice at 12 weeks of age. Cells were incubated under (**A**) Th17 or (**B**, **C**) iTreg skewing condition for 3 days. Cells were restimulated with PMA (50 ng/ml) and ionomycin (1 μg/ml) for 6 h. Cells were harvested and stained with (**A**, **C**) anti-IL-17 or (**B**) anti-Foxp3 antibodies and analyzed by flow cytometry. Values are means ± SD (*n* = 3-4/group), *P < 0.05.

The proportion of IL-17-producing T cells was significantly increased in SLCs from Smad4 tKO NOD compared with WT NOD mice (Figure 4). TGF-β is required for Th17 and iTreg cell differentiation; however, Smad4 is known to be involved in the development of iTreg but not Th17 cells [20, 29]. To investigate the effect of Smad4 deficiency on Th17 and iTreg lineage differentiation in T cells from NOD mice, Teff cells were incubated under Th17 or iTreg skewing conditions. Under the Th17 skewing condition, the IL-17^+^ T cell population was similar between Smad4 tKO NOD and WT NOD T cells (Figure 7A). However under the iTreg skewing condition, Foxp3-expressing cells were decreased in Smad4 tKO NOD compared with WT NOD Teff cells (Figure 7B). Interestingly, IL-17-expressing T cells were increased in Teff cells from Smad4 tKO NOD mice under the iTreg skewing condition compared with WT NOD Teff cells (Figure 7C). These results show that the lack of Smad4 in T cells decreased iTreg differentiation and increased Th17 differentiation.

## DISCUSSION

T cell-specific deletion of TGF-βRII in NOD mice results in severe inflammatory infiltration of multiple organs and death at 3-4 weeks of age, before development of hyperglycemia [30], similar to T cell-specific TGF-βRII deletion in C57BL/6 mice [5, 17]. However, the role of intracellular signaling molecules for TGF-β has not been determined yet in NOD mice. In the present study, we report for the first time that T cell-specific depletion of Smad4 from NOD mice accelerated the development of SS. These mice exhibited typical features of SS, including increased T cell infiltration into lacrimal glands and increased levels of circulating autoantibodies. In addition, the development of diabetes was also accelerated in these mice [31].

It is known that SS is caused by T cell-mediated autoimmune responses to self-antigens on exocrine glands [32]. The mononuclear infiltrates consist predominantly of T cells (CD4 > CD8), with some B cells and plasma cells [2]. We found that Smad4 tKO NOD mice showed enlarged draining lymph nodes and high amounts of lymphocytes with increased granularity compared with WT NOD mice [23, 33]. This suggests that T cells from Smad4 tKO NOD mice have greater proliferative properties. In addition, the proportion and number of activated/memory CD4^+^ and CD8^+^ T cells in draining lymph nodes were increased in Smad4 tKO NOD mice. These results are consistent with phenotype of leukemic LGLs, which are terminal effector memory T cells [25]. As effector memory T cells undergo basal proliferation and quickly exhibit effector responses when stimulated [24], our results suggest that T cells in Smad4 tKO NOD mice might be more active in response to autoantigens.

SS is known to be mediated by inflammatory cytokines, including IL-17 and IFN-γ [34-36], and the TGF-β signal is crucial for Th17 cell differentiation [20]. Therefore, an increase in the proportion of IFN-γ- and IL-17-producing T cells seen in SLCs from Smad4 tKO NOD mice suggests that Smad4-mediated TGF-β signaling is crucial for the development of SS but not for Th17 differentiation. In addition, unlike naïve T cells that require TGF-β and IL-6 for Th17 differentiation, activated or memory T cells can directly differentiate to Th17 under the influence of IL-23 and IL-1β [37]. In fact, we found that the proportion of activated/memory type T cells and transcripts of IL-23 and IL-1β were increased in SLCs from Smad4 tKO NOD mice (Supplementary Figure 3). These results suggest that T cells from Smad4 tKO NOD mice were in a highly activated state compared with WT NOD mice. Since IL-17-producing T cells were increased in Smad4 tKO NOD mice, we examined IL-17 expression under Th17 cell linage differentiation. IL-17-expressing T cells were similar between Smad4 tKO and WT NOD T cells under the Th17 skewing condition, indicating that Th17 differentiation is independent of Smad4. Non-Smad pathways, such as tumor necrosis factor receptor associated factor (TRAF)6-p38/c-Jun N-terminal kinase, and phosphatidylinositide 3-kinase, might be involved in Th17 differentiation. Recently, AKT-mammalian target of rapamycin and mitogen-activated protein kinase pathways were found to contribute to Th17 differentiation [38], and TRAF6 is considered to be involved in Smad4-independent Th17 differentiation [39].

In accordance with previous findings that Smad4 deficiency attenuates TGF-β-mediated *in vitro* polarization of iTreg differentiation [20, 29], we also found that Smad4-deficient NOD T cells had decreased iTreg differentiation. Importantly, IL-17 expression in T cells from Smad4 tKO NOD mice was increased under the iTreg skewing condition. Recently, a study reported that TGF-β1 induces the expression of SET and MYND domain-containing protein 3 (Smyd3) in iTreg cells through the Smad3 pathway [40]. In addition, Smyd3-deficient T cells induced IL-17 expression in the iTreg skewing condition [40]. Therefore, it is possible that Smad4 deficiency decreases translocation of the Smad3/Smad4 complex, and subsequently Smyd3, resulting in the induction of IL-17 expression. Whatever mechanisms are involved, it is clear that the Smad4 signaling pathway is important for regulation of effector T cell response.

Autoimmune diseases can be caused by impaired Treg cell-mediated suppression such as reduction of Treg cell numbers, weakening of Treg suppressive function or resistance of Teff cells to Treg cells [27]. We found that the proportion of natural Treg cells in draining lymph nodes was not changed in Smad4 tKO NOD mice. In addition, there was no significant difference in the mRNA expression of Foxp3 or the suppressive function of Treg cells. Both Smad-dependent and Smad-independent pathways are known to be involved in development and function of Treg cells by TGF-β signaling [41]. A study using T cell-specific Smad2/3 double KO revealed that the Smad pathway is involved in the activation of non-Treg cells and the development of iTreg cells [42]. We found that Teff cells from Smad4 tKO NOD mice were resistant to Treg suppression compared with Teff cells from WT NOD mice, and T cells from Smad4 tKO NOD mice proliferated more than T cells from WT NOD mice upon stimulation *in vitro*. This resistance of Teff cells from Smad4 tKO NOD mice to the suppressive function of Treg cells and TGF-β suggests that Smad4-dependent TGF-β signaling is crucial to develop pathologic Teff cells and SS.

Although T cells are considered as major effectors in SS, many reports have shown that B cells also play a pathogenic role in its development [43, 44]. Exocrine tissue infiltrated with CD4^+^ T cells and dendritic cells produce B cell-activating factors including B cell activating factor and APRIL (a proliferation inducing ligand) [44]. These factors dysregulate B cells, resulting in alterations in subpopulations of peripheral B cells and oligoclonal B cell expansion, and subsequently increase circulating immune complexes and autoantibodies [43]. As serum autoantibodies were increased in Smad4 tKO NOD mice, B cells were clearly activated in these mice. In fact, we found increased proportions of B cells and follicular helper T cells in draining lymph nodes in Smad4 tKO compared with WT NOD mice (Supplementary Figure 4). These results indicate that Smad4 in T cells contributes to the activation and development of autoreactive B cells. How Smad4-deficient T cells contribute to the activation of B cells remains to be studied.

In conclusion, we have shown that disruption of the Smad4 pathway in T cells of NOD mice dysregulates effector T cell activation not by an alteration of Treg function, but by upregulation of Th17, which may contribute to the acceleration and increase of the development of the SS-like symptoms in NOD mice.

## MATERIALS AND METHODS

### Animals

Smad4^fl/+;CD4-Cre^ NOD mice were generated by backcrossing Smad4^fl/fl;CD4-Cre^ (Smad4 tKO) mice in C57BL/6 background [45] with NOD/ShiLtj mice for 7 to 11 generations. Subsequently, Smad4^+/+;CD4-Cre^ (wild-type, WT) and Smad4 tKO NOD mice were generated by heterozygotic brother-sister inbreeding. CD4-Cre recombinase is expressed first in the double-positive thymocytes, thus Smad4 tKO mice do not express Smad4 in either peripheral CD4^+^ T or CD8^+^ T cells [46]. All experiments used age-matched female WT NOD and Smad4 tKO NOD mice. Mice were housed at the animal facility of Lee Gil Ya Cancer and Diabetes Institute (Gachon University, Incheon, Korea), and all experiments were approved by the Institutional Animal Care and Use Committee at Lee Gil Ya Cancer and Diabetes Institute, Gachon University.

### Determination of disease incidence

Disease scoring from 0 to 4 was determined by the visible pathology of each eye (Table 1) on a weekly basis from 6 to 30 weeks of age. Mice were considered to have SS if the sum of pathological scores for both eyes was over 4.0. Any mice that developed diabetes were excluded.

### Reverse transcription-polymerase chain reaction (RT-PCR) and quantitative real-time PCR (qRT-PCR)

Total RNA was extracted using RNAiso Plus (TaKaRa, Otsu, Japan) and cDNA was synthesized using a PrimeScriptTM 1^st^ strand cDNA synthesis kit (TaKaRa). Thermocycling conditions for semi-quantitative RT-PCR were 30 sec at 94°C, 30 sec at 58°C and 30 sec at 72°C for 32-35 cycles preceded by 10 min at 94°C. qRT-PCR analysis was performed by using SYBR Master Mix (TaKaRa) and the CFX384™ Real-Time PCR System (BIO-RAD, Hercules, CA, USA). Relative copy number was calculated using the threshold crossing point (Ct) as calculated by the ΔΔCt calculations. All primer sequences are listed in Supplementary Table 1.

### Measurement of saliva and tear volume

Pilocarpine-stimulated saliva and tear volume was measured as described previously [47]. Briefly, 12-week-old mice were fasted for 5-7 h and then anesthetized with an intraperitoneal (i.p.) injection of ketamine (25 mg/kg body weight; Huons Co., Kyounggi, Korea). Following anesthesia, mice were injected i.p. with pilocarpine hydrochloride (Sigma-Aldrich, St. Louis, MO, USA) at 1 and 5 mg/kg body weight to induce saliva and tear secretion, respectively. Ten minutes after injection, tear volumes were determined using a phenol red thread (Zone-Quick, Menicon, Tokyo, Japan). Once salivation became visible, fluid from the oral cavity was collected by cotton swabs for 10 minutes. The volume was measured and normalized according to body weight.

### Determination of serum autoantibodies

Serum samples were analyzed for autoantibodies against SS-related antigen A (SSA/Ro) and SS-related antigen B (SSB/La) using a commercially available ELISA kit (Alpha Diagnostic International Inc., San Antonio, TX, USA). For detection of anti-nuclear antibodies, NIH 3T3 cells [48, 49] were grown on coverslips in 6-well dishes and fixed with 4% paraformaldehyde. Cells were incubated with serum from 12-week-old Smad4 tKO or WT NOD mice and then stained with anti-mouse IgG-fluorescein isothiocyanate (FITC) antibody (Santa Cruz., Dallas, TX, USA) and 4’,6-diamidino-2-phenylindole (DAPI; Molecular Probes, Invitrogen, Carlsbad, CA, USA).

### Histopathologic analysis

Lacrimal and salivary glands were removed and fixed in 10% neutral-buffered formalin, embedded in paraffin, sectioned at 5-6 μm and stained with hematoxylin and eosin. Images were observed under a Zeiss-LSM light microscope (Zeiss, Munich, Germany). Evaluation of histological score was determined as the number of infiltrated foci as follows: score 0, no infiltrated foci; score 1, less than 1 infiltrated focus; score 2, 2~5 infiltrated foci; score 3, 6~9 infiltrated foci; score 4, over 10 infiltrated foci. Focus was defined as the infiltrated region (0.04 mm^2^) in the section.

### Measurement of cytokine production in lacrimal and salivary glands

Fresh lacrimal and salivary glands were harvested from the mice at 12 weeks of age and lysed in protein extraction buffer (100 mM Tris, pH 7.4, 150 mM NaCl, 1 mM EGTA, 1 mM EDTA, 1% Triton X-100, 0.5% Sodium deoxycholate) containing with 1 mM PMSF, protease and phosphatase inhibitor cocktail (Sigma-Aldrich). After centrifugation, the supernatant was harvested and IFN-γ and IL-17 were measured by LEGEND MAX™ mouse IFN-γ or IL-17a ELISA Kit (BioLegend, San Diego, CA, USA) in accordance with the manufacturer's protocol.

### Analysis of lymphocyte granularity

SLCs were isolated from WT and Smad4 tKO NOD mice at 12 weeks of age and single cell suspensions were smeared on slides. Cells were stained with Wright's-Giemsa and observed under a Zeiss-LSM light microscope. Cellular granularity, which is related to the internal complexity of the scattered cells, was measured by side scatter during flow cytometry. The granularity of gated CD3^+^ T, CD4^+^ T and CD8^+^ T cells was normalized by the mean fluorescence intensity of T cells from WT NOD mice.

### Flow cytometry analysis

SLCs were suspended in 1% heat-inactivated fetal bovine serum (FBS; Gibco BRL, Life Technologies Inc., Grand Island, NY, USA) in phosphate buffered saline. Cells were stained with specific antibodies. Antibodies used are listed in Supplementary Table 2. To measure the proportion of cells producing cytokines, SLCs were cultured in complete RPMI-1640 medium containing 10% FBS and 1% antibiotic-antimycotics (Gibco BRL) with 10 ng/ml phorbol 12-myristate 13-acetate (PMA) and 500 ng/ml ionomycin (Sigma-Aldrich) for 6 h, and then 1 μg/ml brefeldin A (eBioscience, San Diego, CA, USA) was added for 6 h. Cells were stained with surface markers (for CD4 or CD8 T cells), fixed and permeabilized with a Cytoperm/Cytofix Kit (eBioscience), and then stained with specific cytokine antibodies. Cells were analyzed in a FACS LSRII using CellQuest™ Pro Software (BD Biosciences, San Jose, CA, USA). Proportion and absolute counts of specific T cell subsets in SLCs were calculated by multiplying the subject's SLCs by the percentage of the particular T-cell subset obtained by flow cytometry [50].

### T cell isolation

Single cell suspensions were isolated from the spleen and SLCs of WT NOD and Smad4 tKO NOD mice. T cells were enriched using a mouse T-cell enrichment column (R&D System Inc., Minneapolis, MN, USA) and stained with anti-CD4 and anti-CD25 antibodies. Treg cells (CD4^+^CD25^+^) and effector T (Teff; CD4^+^CD25^−^) cells were isolated by the FACS AriaII system (BD Bioscience).

### *In vitro* T cell functional assay

Teff cells were stained with 5 μM carboxyfluorescein succinimidyl ester (CFSE; Invitrogen) and suspended in complete RPMI-1640 medium. CFSE-labelled Teff cells (5 × 10^4^ cells/well) were stimulated with Dynabeads^®^ Mouse T-Activator CD3/CD28 (Life Technologies, 2 μl/well) for 72 h in the presence of Treg cells at various ratios on 96-well round-bottom plates (BD Biosciences). For inhibition of TGF-β1, CFSE-labelled Teff cells were stimulated with Dynabeads^®^ Mouse T-Activator CD3/CD28 for 72 h with or without recombinant human TGF-β1 (PeproTech. INC., Rocky Hill, NJ, USA). Then, CFSE fluorescence intensity was measured by FACS LSRII to determine the proliferation of Teff cells.

### *In vitro* T cell differentiation

Teff cells (3 × 10^5^ cells/well) were cultured in complete RPMI-1640 medium and stimulated with Dynabeads^®^ Mouse T-Activator CD3/CD28 (8 μl/well) for 72 h along with cytokines and neutralizing antibodies for the desired polarization as follows: human-IL-6 (100 ng/ml; PeproTech. INC.), mouse-IL-23 (10 ng/ml; Biolegend), human-TGF-β (5 ng/ml), anti-IL4 (10 μg/ml; Biolegend) and anti-IFN-γ (10 μg/ml; Biolegend) for Th17; mouse IL-2 (10 ng/ml), human-TGF-β (2 ng/ml), anti-IL4 (10 μg/ml) and anti-IFN-γ (10 μg/ml) for inducible Treg (iTreg) cell polarization. Cells were restimulated with PMA (50 ng/ml), ionomycin (1 μg/ml) and brefeldin A (1 μg/ml) for 6 h. Cells were harvested and stained with anti-IL-17 or anti-Foxp3 antibodies and analyzed by flow cytometry.

### Statistical analysis

Data are presented as means ± standard deviation (SD). Statistical significance of the difference between two groups was analyzed by unpaired Student's *t*-test. The value of statistical significance was set at *P* value < 0.05.

## SUPPLEMENTARY MATERIALS FIGURES AND TABLES


