# Supplementary material for: Smad4 in T cells plays a protective role in the development of autoimmune Sjögren's syndrome in the nonobese diabetic mouse
Source: Oncotarget. 2016 Nov 17;7(49):80298–312. doi: 10.18632/oncotarget.13437 (PMC5348321; doi:10.18632/oncotarget.13437)
Supplement: Supplementary file 1 [file oncotarget-07-80298-s001.pdf]

Smad4 in T cells plays a protective role in the development of autoimmune Sjögren's syndrome in the nonobese diabetic mouse

Supplementary Material

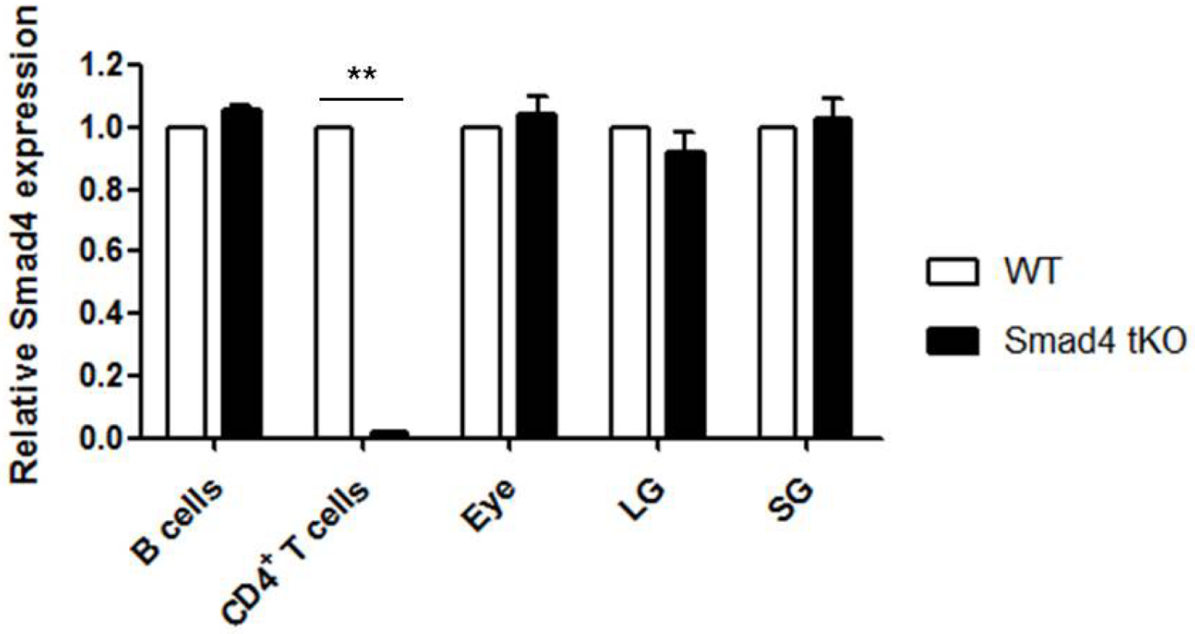

Supplementary Figure 1. Smad4 mRNA is not expressed in T cells from Smad4 tKO NOD mice. Total RNA was isolated from splenic B cells, $CD4^+$ T cells, eye, lacrimal glands and salivary glands of Smad4 tKO and WT NOD mice at 5-6 weeks of age. The expression of Smad4 mRNA was analyzed by qRT-PCR. Values are expressed as the relative fold-change of the level expressed in WT NOD mice. Data are mean ± SD (n = 3/group). *P < 0.05, **P<0.01.

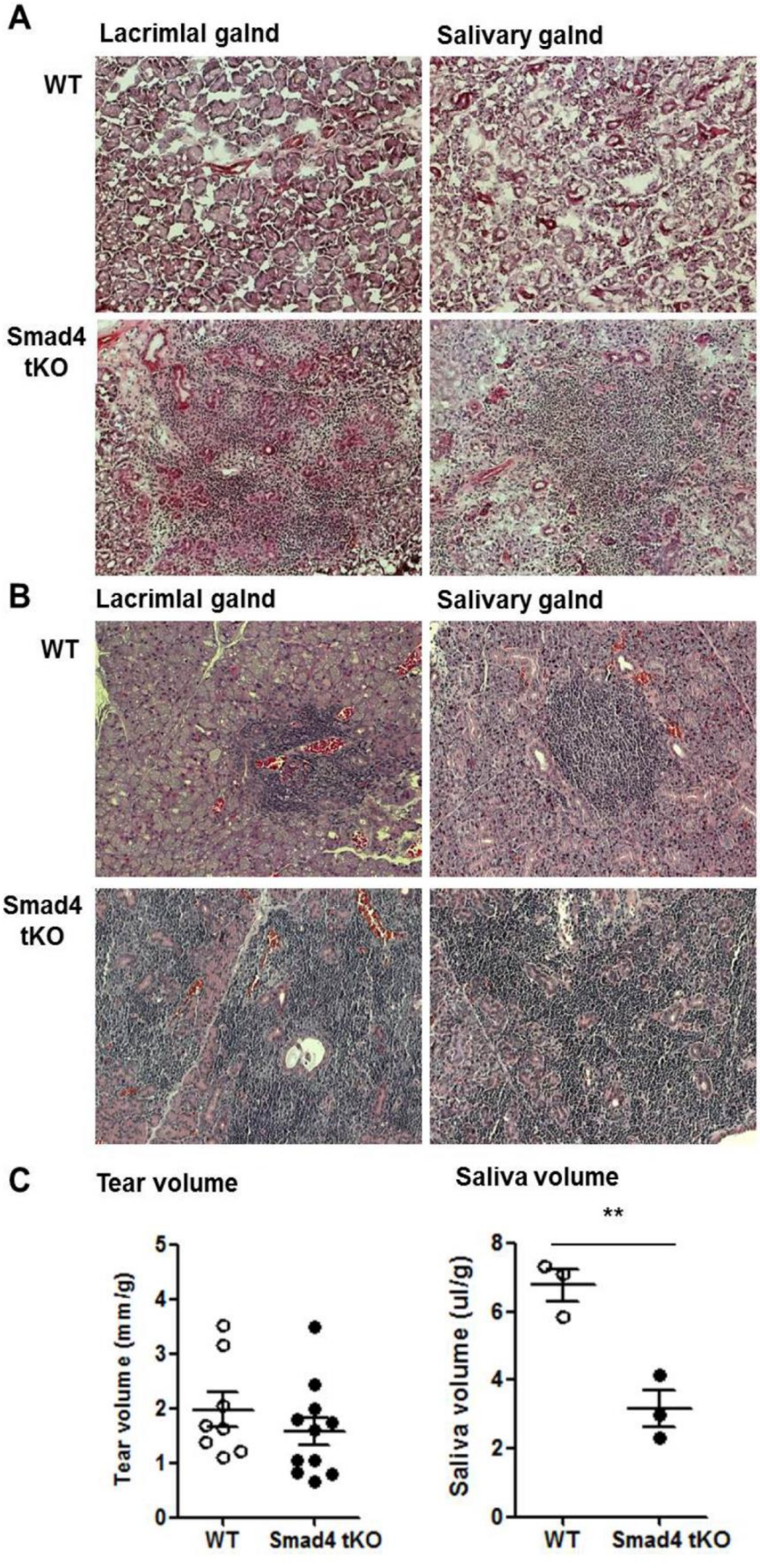
A
Lacrimlal galnd
Salivary galnd
WT
Smad4 tKO
B
Lacrimlal galnd
Salivary galnd
WT
Smad4 tKO
C
Tear volume
Saliva volume
Tear volume (mm/g)
0
1
2
3
4
5
WT
Smad4 tKO
**
Saliva volume (ul/g)
0
2
4
6
8
WT
Smad4 tKO

Supplementary Figure 2. Lymphocyte infiltration in lacrimal and salivary glands is increased and saliva volumes are decreased in Smad4 tKO NOD mice at 20 weeks of age. (A-B) Sections of lacrimal and salivary glands from (A) 12-week old and (B) 20-week old mice were stained with hematoxylin and eosin. (C) Tear and saliva volumes from 20-week-old mice normalized by body weight.

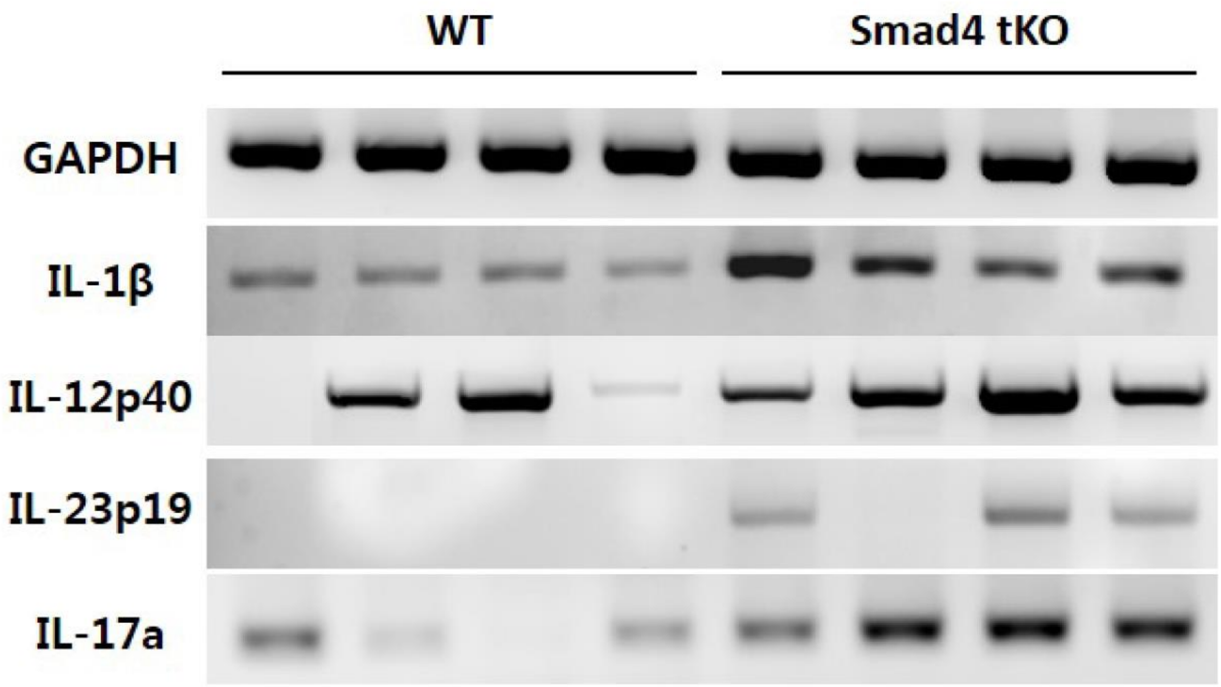

Supplementary Figure 3. Expression of inflammatory cytokines is increased in SLCs from Smad4 tKO NOD mice. Total RNA was isolated from SLCs of female Smad4 tKO and WT NOD mice at 12 weeks of age. The expression of mRNA for IL-1β, IL-12, IL-23 and IL-17 was analyzed by semi-quantitative RT-PCR.

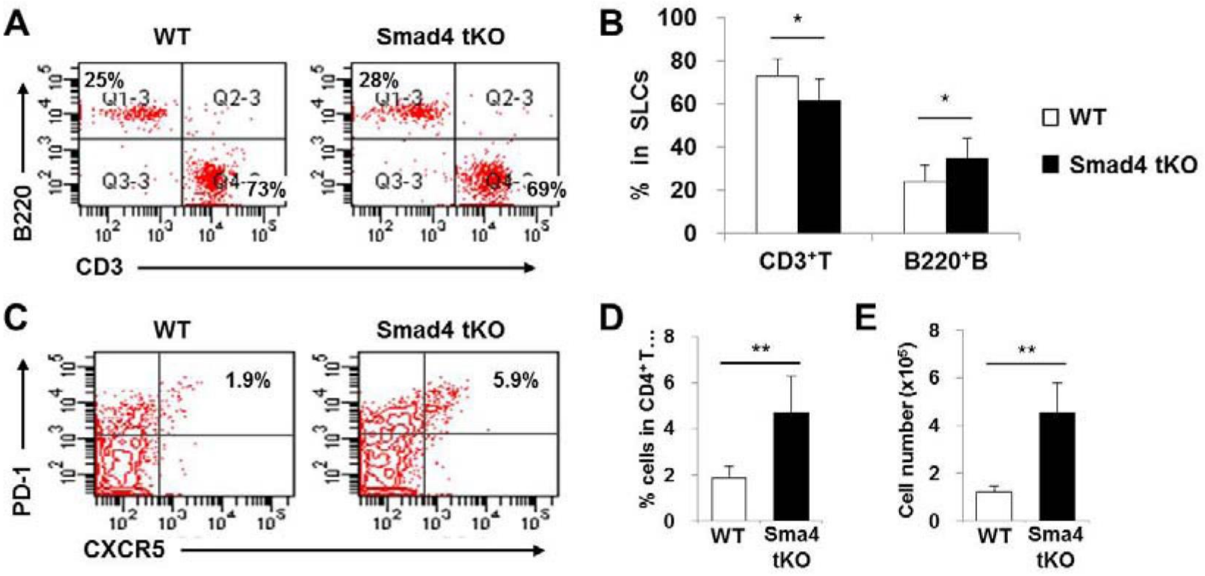

Supplementary Figure 4. B cells and follicular helper T cells are increased in SLCs from Smad4 tKO NOD mice. SLCs were isolated from 12-week old female Smad4 tKO and WT NOD mice. (A) SLCs were stained with anti-CD3 and anti-B220 antibodies and analyzed by flow cytometry. (B) The proportion of T and B cells was determined as a percent in total SLCs. (C) SLCs were stained with anti-CD4, anti-CXCR5 and anti-PD-1 antibodies and analyzed by flow cytometry. (D) The proportion and (E) cellular numbers of the follicular helper T (Tfh) cell population was determined as a percent in $CD4^+$ gated T cells. Values are means ± SD (n = 5-6/group), $^*P<0.05$, $^{**}P<0.01$ compared with the WT group.

Supplementary Table 1. List of primers for RT-PCR

Gene symbol	Forward primer	Reverse primer
Smad4	GGCCAGTTCACAATGAGCTT	CCATCCACAGTCACAACAGG
IL-1β	ATGGCAACTGTTCCTGAACTC	CAGGACAGGTATAGATTCTTT
IL-12p40	ATGTGTCCTCAGAAGCTAACCA	CTAGGATCGGACCCTGCA
IL-17a	GTCCAAACACTGAGGCCAAG	CTTCATTGCGGTGGAGAGTC
IL-23p19	ATGCTGGATTGCAGAGCAGTA	TTAAGCTGTTGGCACTAAGGG
GAPDH	TGAGCCCTTCCACAATGCCA	AGTGCCAGCCTCGTCCCGTA
*T-bet	AATCGACAACAACCCCTTTG	CGAGGGGACACTCGTATCAA
*Gata3	CTACCGGGTTCGGATGTAAGTC	GTTCACACACTCCCTGCCTTCT
*RORγt	TCTACACGGCCCTGGTTCT	ATGTTCCACTCTCCTCTTCTCTTG
*Stat3	TCCAAGCAGTGATTTCTCCCTAGAAC	GAGAGGTACTTCTGGTACCTTTTTCC
*IL-4	ACAGGAGAAGGGACGCCA	GAAGCCCTACAGACGAGCTCA
*IL-6	TCCAGTTGCCTTCTTGGGACTGAT	AGCCTCCGACTTGTCAAGTGGTAT
*IL-17	GTGTCAATGCGGAGGGAA	TTCAGGACCAGGATCTCTTGCT
*IFN-γ	CGGCACAGTCATTGAAAGCCTA	GTTGCTGATGGCCTGATTGTC
*Foxp3	GGCCCTTCTCCAGGACAGA	GCTGATCATGGCTGGGTTGT
*Smad4	AATTGCCTCACCACCAAAAC	CACGTGAGCACAGTGCGTTTA
*TGF-β	GATTCAGCGCTCACTGCTCTT	GGGCTGATCCCGTTGATTT
*IL-10	GGCGCTGTCATCGATTTCTC	TCTTGGAGCTTATTAAAATCACTCTTCA
*Cyclophilin B	TGGAGAGCACCAAGACAGACA	TGCCGGAGTCGACAATGAT

* Primers for qRT-PCR

Supplementary Table 2. List of antibodies for this study

Antibody	Source (Catalogue No.)	Dilution	Usage
Anti-Mouse CD3-PE	BD Pharmingen™(553064)	1:100	FACS
Anti-Mouse CD4 PE-Cyanine7	eBioscience (25-0041-81)	1:100	FACS
Anti-Mouse-CD4-FITC	Biolegend (100405)	1:100	FACS
Anti-Mouse CD25 APC	eBioscience (17-0251)	1:100	FACS
Anti-Mouse CD16/CD32 (Fc Block)	BD Pharmingen™(553142)	1:100	FACS
Anti-Mouse-CD4-APC-Cy7	BD Pharmingen (552051)	1:100	FACS
Anti-Mouse-CD8a–Pacific blue	BD Pharmingen (558106)	1:100	FACS
Anti-Mouse CD44-APC	BD Pharmingen (559250)	1:100	FACS
Anti-Mouse-CD62L-FITC	BD Pharmingen (553150)	1:100	FACS
Anti-Mouse/Rat-Foxp3-PE-Cy7	eBioscience (25-5773)	1:100	FACS
Anti-Mouse-IL-4-PE	BD Pharmingen (554435)	1:100	FACS
Anti-Mouse-IL-17-PE	BD Pharmingen (559502)	1:100	FACS
Anti-Mouse-IFN-γ-PE-Cy7	BD Pharmingen (557649)	1:100	FACS
Anti-Smad4 antibody	Santa Cruz (sc-7966)	1:100	FACS
FITC-conjugated anti-mouse IgG	Santa Cruz (sc-2010)	1:100	IFA, FACS
